# Feasibility, Usability, and Customer Satisfaction of the Tele-COVID19 Project, Sicilian Model

**DOI:** 10.3390/medicina58081110

**Published:** 2022-08-16

**Authors:** Giuseppa Maresca, Smeralda Anchesi, Lilla Bonanno, Alessia Bramanti, Lara Carnazza, Vincenzo Cimino, Francesco Corallo, Viviana Lo Buono, Fabio Mauro Giambò, Desiree Latella, Nicholas Parasporo, Maria Cristina De Cola

**Affiliations:** 1 IRCCS Centro Neurolesi Bonino-Pulejo, 98124 Messina, Italy; 2 Department of Medicine, Surgery and Dentistry, Medical School of Salerno, University of Salerno, 84100 Salerno, Italy

**Keywords:** COVID-19, psychological tele-counselling, telehealth, telemedicine, tele-monitoring

## Abstract

*Background and Objectives*: In March 2020, COVID-19 pandemic affected the world. All countries, to limit viral transmission, imposed quarantine. This emergency exerted personal, social, economic, and psychological impact on people. For health systems, was needed to create alternative care pathways. Telemedicine can be helpful to reduce isolation, provide health care services, and monitor virus infections. Italian regions, including Sicily, have activated telemedicine services for management of patients with COVID-19. Objective: The purpose of study is to describe a Sicilian telemedicine model for management of COVID-19 patients, showing results on feasibility, usability and quality of service and patient satisfaction. *Materials and Methods*: This is a descriptive exploratory study on a telemedicine service for residents in Messina infected by COVID-19. It included monitoring of vital signs and specialist consultations (i.e., doctor, psychologists, social workers, and nutritionist biologists). *Results*: More than twenty percent (23.8%) of participants used tele-monitoring and tele-counselling services; 14.3% were only telemonitored. Participants judged positively telemedicine service (30% were quiet and 50% were very satisfied), as well as tool (70% were quiet and 10% were very satisfied). Telemonitoring had a low agreement (10% were slightly satisfied and 50% were neutral); tele-counselling had a high rate of satisfaction (40% quiet and 60% were very satisfied). *Conclusions*: This study showed that telemedicine model for Sicilian population affected by COVID-19 was feasible, easy to use and appreciated by patients. Our promising results allow us to assume that if in Sicily there was a return of the emergency, we would be ready to manage it This system can be the solution to remote management of these patients, to reduce isolation, provide health services, and monitor virus infections. The use of this technology should encourage future research to change the health care system and provides opportunities to ensure health and care for oneself and others.

## 1. Introduction

In December 2019, China was affected by Severe Acute Respiratory Syndrome Coronavirus 2 (SARS-CoV-2). This virus reached Italy at the end of February 2020, hitting primarily the Lombardy region, and then spreading throughout the territory [1]. As with other respiratory pathogens, the virus could be transmitted from human-to-human, so isolation represented the best way to contain this epidemic. On 11 March, the World Health Organization (WHO) declared the “pandemic state” leading governments of the mainly affected countries to impose strict confinement on their citizens. In Italy, on 8 March, the government had established extraordinary measures to limit viral transmission, imposing the quarantine of the population since March 10 [2]. This event exerted a strong impact on the life of every individual on many levels (personal, work, social, economic, and psychological), the strongest lived since the Second World War certainly. Thus, new rules had been imposed, such as working from home and closing schools, shops, and any not essential service, in order to slow down the spread of the contagion and prevent the collapse of healthcare systems [3]. Because of the great impact of these measures on the population’s general health, it has been necessary to act on treatment programs to support health workers involved in the care of COVID-19 patients [4]. In this new emerging challenge for health systems worldwide, where social distance is carried out to reduce the spread of infection, there is an emerging need to create new and innovative care pathways, not only in hospitals but especially at patient’s home. In this background, telemedicine can be a convenient solution to face the social distancing imposed by the pandemic, as seen in Singapore during COVID-19 with the use of chatbots, a common feature used by most government agencies, which provides immediate access to information [5,6]. Advances in technology and communication have influenced the development of telemedicine and telecare solutions for home care, also with the aim of controlling the health costs of the population [6]. Telehealth using videoconferencing has been an established model of healthcare delivery for clinical consultations for many years, although it has enjoyed only a limited uptake, due to several barriers, such as issues with technically challenged staff and resistance to change, cost, patient’s age, and level of education [7]. Thus, we can imagine that telemedicine-based services could reduce the sense of isolation due to quarantine, provide for inaccessible health care services, and support people’s needs. They could also be used to monitor the virus infections, in addition to comorbidities, such as heart failure, diabetes, and chronic obstructive pulmonary disease, resulted to be risk factors for mortality due to COVID-19 [8]. To this purpose, healthcare facilities are providing clinical services at a distance, where possible. This keeps vulnerable multi-morbid patients at high risk of COVID-19 away from hospitals, primary care clinics and other patients who may be infected with COVID-19 [9]. In Italy, the prevalence of COVID-19 infections has not been homogeneous, occurring especially in the northern regions. However, all Italian regions have activated telemedicine services for the remote management of patients infected with COVID-19 who did not require hospitalization. Sicily in March 2021, with its low number of 3443 registered cases (986 positives of which 72 hospitalized with symptoms and 914 in home isolation, 2183 cured and 274 deceased), has also moved in this direction. Thus, the Sicilian government and the Istituto di Ricovero e Cura a Carattere Scientifico (IRCCS) Centro Neurolesi “Bonino-Pulejo” of Messina designed a telemedicine service called “TeleCOVID-19”, which included the tele-monitoring of vital parameters in all infected subjects, together with services of telepsychology, physician and physiotherapist tele-counselling, speech therapy, and even nutritional support and social service assistance. Other studies have also highlighted the need to apply telemedicine to provide continuity in the care of patients; however, most of the research has focused on previous diseases or on the field of rehabilitation. These studies have focused on only one aspect of health that could be managed remotely [10,11,12,13]. The purpose of this study is to describe a Sicilian telemedicine model for management of COVID-19 patients, showing preliminary results on the feasibility of the telemedicine service, in relation with the type of telemedicine service received and the virus symptoms, and the patient’s usability of the technology. Moreover, we also investigated the quality of the service and the patient satisfaction.

## 2. Materials and Methods

### 2.1. Study Design and Setting

**Figure 1:**
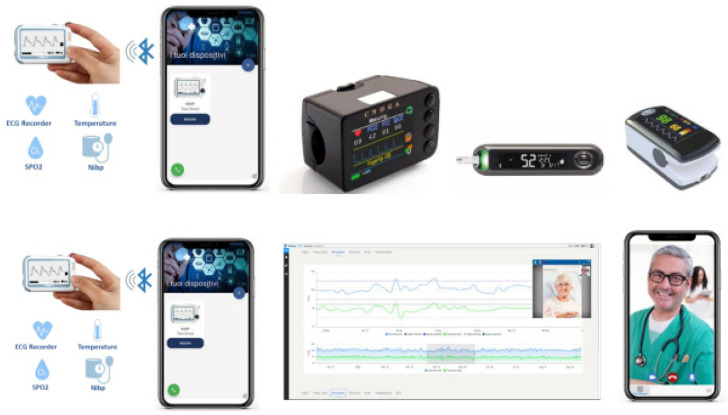
Tools used for telemedicine service.

This is a descriptive exploratory study. We provided a telemedicine service for residents in the city of Messina (Sicily) infected by COVID-19. This service included remote surveillance and socio-territorial assistance combined with health services (Figure 1). The inclusion criteria were: COVID-19 infection, age over 18 years. The exclusion criteria were hospitalization, visual and auditory impairment. This study involved a multidisciplinary team consisting of all specialists who had the expertise to manage COVID from a clinical and rehabilitation perspective.

Each patient who chose to enroll in this service was referred by Provincial Health Care Providers (ASP). For each enrollment, operators went directly to the users’ homes and delivered a tablet equipped with all the accessories for monitoring. During the delivery was made a brief training for the proper use of the platform.

### 2.2. Study Population

Although eighty-two COVID-19 infected adults were eligible for being included in this study, forty subjects refused to participate (Table 1). Thus, 42 adults (57.76% male, age 48.38 ± 21.96 years) living in urban (57.14%) or rural (42.86%) areas were recruited into this study protocol through USCA (Special Continuity Care Units) after signing an informed consent. Informed consent was obtained from all subjects involved in the study.

About 45.2% of them were asymptomatic, 26.2% presented symptoms, and 28.6% had paucity of symptoms. A more detailed description of the sample characteristics is provided in Table 2. The study was approved by the Regional Ethics Committee.

### 2.3. Assessment

To measure the service feasibility, we considered the proportion of subjects who used the complete telemedicine program on the total participants, as well as the rate of abandoning, for each type of symptomatology. We also considered the reasons for refusal of service.

At the end of the telemedicine program, the System Usability Scale (SUS) and a brief questionnaire were anonymously administered through an online survey, in order to evaluate the usability and feasibility of the system and its satisfaction. The SUS is a 10-item scale giving a global view of subjective assessments of usability, which score ranged from 0 to 100 [14]. The brief questionnaire included four 1–5 Likert scales, where 1 = totally not satisfied and 5 = totally satisfied, for evaluating the acceptance of: (i) the global service; (ii) the telemedicine tool; (iii) the telemonitoring service; (iv) the tele-counselling service. Finally, to evaluate the quality of the telemedicine service, we considered the number of tele-counselling activated, and the number of tele-counselling sessions canceled, on the total participants, for each specialty. Regarding the test scoring, some calculation steps were performed. The score was obtained by subtracting 1 from the score of each odd answer and subtracting the score of each even answer from 5. The total score was obtained by adding these new scores and multiplying by 2.5. Since 100 is the maximum score, the average score is 68.

### 2.4. Statistical Analysis

Descriptive analysis was reported on demographic and clinical variables. Continuous variables were expressed in mean ± standard deviation, whereas categorical variables in frequencies and percentages. Since the small dimension of the sample nonparametric tests were used. Notably, the Mann–Whitney U test for independent samples was used to compare continuous variables, whereas the chi-squared test or the Fisher’s exact test were used to assess for statistical differences in proportions.

Correlations between variables were assessed by means of the Spearman’s rank correlation coefficient or the point-biserial correlation coefficient when one variable was dichotomous. Statistical analysis was performed by using the 3.5.0 version of the open-source software R (Institute for Medical and Biomedical Education, St. George’s, University of London, UK), and a *p* < 0.05 was considered as statistically significant.

## 3. Procedure

### 3.1. The Telemedicine Service

The telemedicine service included daily monitoring of vital signs, in addition to clinical consultations with a medical doctor (i.e., neurologist, cardiologist, pneumologist, radiologist, endocrinologist, physiatrist). Upon the user’s request, specialist tele-counselling services were available (i.e., psychologists, social workers, and nutritionist biologists). Moreover, to deal with the decrease in walking and speech abilities due to therapy interruption, subjects with a neurological disease could be also followed by skilled physiotherapists and speech therapists. In the case of respiratory symptoms worsening, the telemedicine system can be used to transmit in real-time the chest radiography performed through a portable device for identification and follow-up of lung abnormalities.

Tele counselling was provided by means of audio-video conference sessions of about 60 min. When the subject demanded a specialist consultation, an operator scheduled the appointment, replying with an email of confirmation.

The telepsychology service has focused on the management and resolution of symptoms resulting from COVID-19 pandemic. Interventions were divided into two levels. The first level was empathic listening, which provided reassurances and suggestions with the purpose of alleviating the psychological distress caused by the pandemic and often resolved in a single telephone session. The second-level intervention involved the management and taking charge of patients who declared psychological changes. Thus, there were psychotherapeutic treatments connected or exacerbated by the emergency, in order to facilitate the reduction of the psychological distress and overcome the traumatic event, in addition to acquiring emotional skills useful for dealing with the post-emergency.

The tele-nutrition service provided individual nutritional programs according to anthropometric measurements, aimed to re-establish a normal nutritional status and improve the quality of the food consumed.

The tele-counselling with the social worker was aimed to bureaucratically support the subjects, providing information regarding territorial resources available or acting as mediator between the patient/caregiver and the general practitioner, in order to prevent and reduce the isolation due to a sense of abandonment from the authorities.

### 3.2. The Telemedicine Platform

The telemedicine platform consists of three main parts: the software, which provides customized access by staff category, the devices for the detection and transmission of vital parameters located at the patient’s home, and a data storage system in the Cloud securely hosted on external servers. The set of telemedicine devices also includes a portable X-Ray, which is located in the telemedicine head office and allows the transmission of chest radiography at the patient’s home using the Digital Imaging and Communications in Medicine (DICOM) standard (at the IRCCS).

Through a series of hardware and software interfaces based on the Bluetooth Low Energy (BLE) wireless communication protocol, the system communicates with the different devices and acquires their data, allowing for more functional low-range communication. Notably, once the measurement is detected, it is transferred to the tablet, which, in turn, sends it to the platform by mobile Internet connection (3G/4G). The devices collect several types of data according to the subjects’ symptomatology. Thus, for the tele-monitoring of patients without symptoms, the device records blood oxygenation and pressure, heart rate, and body temperature, whereas in the tele-monitoring of a symptomatic patient, the device also records hemodynamic, blood gas parameters, glycaemia and flow capacity and volume of the lungs. When a measurement is outside the normal range, an alarm is immediately generated. An operator located at the center checks the report and follows the protocol designed for that situation. Any team member can enter the telemedicine systems by using personal access credentials. According to their own role, the team member can only monitor the health of patients (e.g., operator), or to visualize the measurement of parameters both in real-time and remote (e.g., technician, doctor).

Data transmission exploits secure communication channels in line with health information standards, such as Health Level Seven (HL7) and Fast Healthcare Interoperability Resource (HL7-FHIR), to enable the electronic exchange of health data in both clinical and administrative environments, ensuring the flow of data between different systems, including web-based applications.

## 4. Results

### 4.1. Telemedicine Service Feasibility

**Figure 2:**
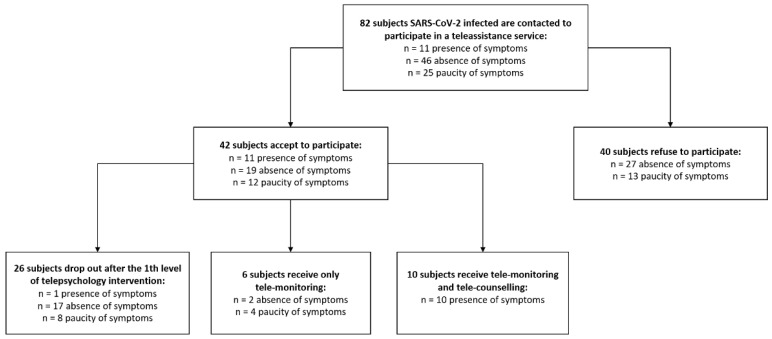
Flow-chart describing participants’ selection procedure.

Out of the 42 participants, 23.8% used both tele-monitoring and tele-counselling services, whereas 61.9% abandoned the service after the first level intervention of telepsychology, and the remaining 14.3% were only tele-monitored (Figure 2).

Symptomatic subjects were mainly men (72.7%), living in urban areas (63.6%), and meanly aged 49.4 ± 14.2 years. About 90.9% of them used the complete telemedicine service, i.e., tele-monitoring and tele-counselling with specialists (1st and 2nd levels), versus no usage from the other categories (χ^2^(2) = 36.99; *p* < 0.001). Notably, the rate of abandoning after the first telepsychology session was significantly higher (χ^2^(2) = 19.25; *p* < 0.001) in subjects without symptoms (89.5%) than in subjects with symptoms (9.1%) and in subjects with a paucity of symptoms (66.7%). No differences in gender, age and area of residence emerged when comparing subjects by symptomatology as shown in Table 1.

### 4.2. Patient Satisfaction and Usability

Participants positively judged the telemedicine service (30% were quite satisfied and 50% were totally satisfied) as well as the telemedicine tool (70% were quite satisfied and 10% were totally satisfied). The service of monitoring had a low agreement (10% were slightly satisfied and 50% were neutral), contrary to the tele-counselling, which got a high rate of satisfaction (40% quite satisfied and 60% were totally satisfied).

The mean SUS score of 81.4 ± 7.15 indicated a high subjects’ usability of the system, which seems to be moderately correlated with age (r = −0.40) and poorly correlated with gender (r = 0.13). We found a moderate correlation between SUS score and the satisfaction of the tele-counselling service (r = 0.43).

### 4.3. Quality of the Service

Telepsychology was the telecounselling service most used. Indeed, about 61% of participants who completed the second-level intervention of tele-psychology made a request for further psychological telecounselling. Moreover, the psychologists provided cognitive rehabilitation to about 20% of the participants, and support to 15% of their caregivers. However, we also registered telecounselling with other specialists. Indeed, about 30% of participants made a request for social worker consultation, 60% for logopedy consultation, 50% for nutritionist biologist consultation, 10% for endocrinology consultation. No participant canceled a session of tele-counselling, and the rate of canceling was 0%. Finally, cardiology consultation was provided for 50% of subjects, on the basis of tele-monitoring alarms, whereas the radiology tele-counselling was provided for 20% of subjects, upon request of a pneumologist. No participant was hospitalized during the provision of the telemedicine service.

## 5. Discussion

In this work, the main objective was to make a service such as telemedicine more usable at such a critical time, starting not from clinical pathways but precisely from the needs of patients at home. Usability, flexibility, and satisfaction were the indicators provided by the patient to understand more quickly what were the most immediate methods of making decisions about the citizen’s health. In particular, usability was used to test how well the tools could be used; flexibility indicated how well the patient could maintain the same trust in a different context towards the doctor; and finally, satisfaction indicated the quality of the service outcome. The results of this work, taking these aspects into account, were very encouraging, especially because the patient was always able to remain the center of attention in the project. We understand that the results may still be insufficient to make certain claims. However, the statistical survey, the little literature regarding telemedicine in the COVID era, has allowed us to try to build an ad hoc model for taking care of patients at home that could be employed even after the pandemic. The COVID-19 emergency is affecting the whole world, with a growing burden on health systems. For this reason, it was necessary to create alternative care, especially in the patient’s home. To the best of our knowledge, this is the first study that aims to verify the feasibility of a telemedicine model aimed at the Sicilian population affected by COVID-19. Our results show that the telemedicine service offered was feasible, easy to use and appreciated by patients (see Table 1). The effectiveness of telemedicine has been widely demonstrated and discussed and in many cases, the data have shown that the model is similar to the treatment that can be provided in a hospital or day hospital [15,16]. However, as has been the case in all health care systems, telemedicine services have had to adapt to cope with the remote health emergency for COVID-19 patients [17,18,19].

This type of system can be the solution to reduce the sense of isolation due to quarantine, to provide otherwise inaccessible health services and also to monitor virus infections in addition to comorbidities [8]. In this sense, our study, the first in Sicily, has provided encouraging data to ensure healthcare during such an emergency period. In fact, the Telecovid service, in addition to having obtained a high level of satisfaction, was suitable for the population and was aimed at those who really needed it; as asymptomatic patients, after the first consultation or after the remission of symptoms, were “discharged from the service”. In our sample, the telemedicine service was considered very satisfactory in terms of quality and general organization. The other Italian regions have also promoted telemedicine services, obtaining a lot of feedback [20,21], but in our model, unlike other projects, we have reported a teleservice model, already used for other types of patients [22,23].

The originality of the project was the multidisciplinary nature of the service; in fact, the management of patients affected by COVID-19 involved several professional figures.

As other studies have also demonstrated [24,25], psychological counselling was the most requested service among those offered by the project. Psychologists within this service were the most requested. The request for the interview with the psychologist was crucial considering the effects that the virus has had on the world population [26,27]. In addition, psychological treatment was offered, not only to the patient, but also to the caregiver, who had to manage the symptoms of his relative and the fear of contagion in solitude, due to social distancing. It is our opinion that the pandemic has forced even the most skeptical to come to terms with the new communication technologies, stimulating an important reflection on the indispensable conditions for the setting up and profitable use of an interactive environment. In this regard, several international associations, including the American Psychological Association (APA), have disseminated the general, technical and deontological guidelines for the effective use of telecommunications tools in the field of psychological interventions [28,29,30].

Another relevant data point that emerged from our study is the high level of patient satisfaction; in fact, it emerged that all the services offered were well accepted, except for telemonitoring (perhaps, detecting the parameters three times a day was a poorly interactive and stimulating activity). In addition, we can assume that the service offered by us has contained the impact of COVID-19 pandemic, thanks to the characteristics of the type of service offered, including the possibility of being managed and monitored 24 h a day and having all the devices available (including the radiological survey), has made the subjects feel safer.

Regarding the limitations of the study, the model should have been extended to a larger number of patients and with a greater variety of pathologies. In addition, due to the lack of predetermined guidelines, accuracy in patient care was not high.

The experience of this model should encourage future research to change the health care system; indeed, COVID-19 has changed lifestyles in many contexts, including health care. The use of technology, as we have shown, provides opportunities to ensure not only health but also care for oneself and others.

Our promising results allow us to assume that if there was a return of the emergency in Sicily, we would be ready to manage it. In fact, it is important to emphasize that this experience does not have an exclusively situational value (COVID-19 period), but could be generalized in future health emergencies of other kinds.

## Figures and Tables

**Table 1:** Characteristics of subjects who did not participate in the Tele-COVID19 project.

	All	Men	Women	*p*-Value
Subjects, N (%)	40 (100)	17 (42.50)	23 (57.50)	-
Age, mean ± SD (years)	46.45 ± 22.58	37.70 ± 21.26	52.91 ± 21.73	0.10
Area of residence, N (%) Urban Rural	23 (57.5) 17 (42.5)	12 (70.59) 5 (20.41)	11 (47.83) 12 (52.17)	0.26
Reason, N (%) Useless Personal issues Diffidence Other reasons	3 (7.5) 3 (7.5) 32 (80) 2 (5)	1 (5.88) 2 (11.77) 13 (76.47) 1 (5.88)	2 (8.69) 1 (4.35) 19 (82.61) 1 (4.35)	0.90

**Table 2:** Participant’s description by symptomatology.

	Presence	Absence	Paucity	*p*-Value
Gender, N (%) Male Female	8 (72.73) 3 (27.27)	11 (57.89) 8 (42.11)	4 (33.33) 8 (66.67)	0.15
Age, mean ± SD (years)	49.36 ± 14.19	43.05 ± 22.89	55.92 ± 25.38	0.28
Area of residence, N (%) Urban Rural	7 (63.64) 4 (36.36)	13 (68.42) 6 (31.58)	4 (33.33) 8 (66.67)	0.14
Telemedicine program, N (%) Only 1th level telepsychology Only tele-monitoring Complete *	1 (9.1) 0 (0.0) 10 (90.9)	17 (89.5) 2 (10.5) 0 (0.0)	8 (66.7) 4 (33.3) 0 (0.0)	<0.001 0.06 <0.001

* Tele-monitoring and tele-counselling with specialists (1th and 2th levels).

## Data Availability

Not applicable.
